# Outbreak Linked to Morel Mushroom Exposure — Montana, 2023

**DOI:** 10.15585/mmwr.mm7310a1

**Published:** 2024-03-14

**Authors:** Heather Demorest, Rachel Hinnenkamp, Maggie Cook-Shimanek, Alyssa N. Troeschel, Michael Yeh, Thao-Phuong Christy Hallett, David Kuai, Johnni Daniel, Andrea Winquist

**Affiliations:** ^1^Gallatin City-County Health Department, Bozeman, Montana; ^2^Montana Department of Public Health and Human Services; ^3^Division of Environmental Health Science and Practice, National Center for Environmental Health, CDC.

**Summary**

**What is already known about this topic?**

Although morel mushrooms are generally considered edible, rare cases of illness have been reported after consumption; little is known about the human health effects of morels. During March–April 2023, a total of 51 persons reported gastrointestinal illness after dining at a Montana restaurant; two patients died.

**What is added by this report?**

A case-control study identified morel mushrooms as the likely outbreak source. Consumption of raw morels was more strongly associated with illness than was consumption of cooked or partially cooked morels.

**What are the implications for public health practice?**

This outbreak investigation highlights the importance of prompt cross-agency communication, collaboration, and the use of epidemiologic studies to guide outbreak investigations. Morel mushrooms should be cooked before eating to mitigate potential toxic effects.

## Abstract

During March–April 2023, a total of 51 persons reported mild to severe gastrointestinal illness after eating at restaurant A in Bozeman, Montana. The outbreak resulted in multiple severe outcomes, including three hospitalizations and two deaths. After an inspection and temporary restaurant closure, the Montana Department of Public Health and Human Services and Montana’s Gallatin City-County Health Department collaborated with CDC to conduct a matched case-control study among restaurant patrons to help identify the source of the outbreak. Consumption of morel mushrooms, which are generally considered edible, was strongly associated with gastrointestinal illness. A dose-response relationship was identified, and consumption of raw morel mushrooms was more strongly associated with illness than was consumption of those that were at least partially cooked. In response to the outbreak, educational public messaging regarding morel mushroom preparation and safety was shared through multiple media sources. The investigation highlights the importance of prompt cross-agency communication and collaboration, the utility of epidemiologic studies in foodborne disease outbreak investigations, and the need for additional research about the impact of morel mushroom consumption on human health. Although the toxins in morel mushrooms that might cause illness are not fully understood, proper preparation procedures, including thorough cooking, might help to limit adverse health effects.

## Introduction

On April 18, 2023, the Montana Department of Public Health and Human Services (MTDPHHS) and health departments in Gallatin and Broadwater counties were notified of two persons who experienced severe nausea, vomiting, and diarrhea after separately dining at restaurant A in Bozeman, Montana on April 17. One restaurant patron was hospitalized and later died; the second died hours after being discharged from a hospital emergency department. Both persons experienced symptom onset within 60 minutes of their meal. These reports prompted an immediate restaurant inspection on April 18 by the Gallatin City-County Health Department (GCCHD) and a temporary closure of restaurant A. On the basis of the rapid onset of symptoms in the hospitalized patient, the provider suspected a foodborne toxin as the source of illness and sought clinical consultation from Rocky Mountain Poison and Drug Safety (https://www.rmpds.org/) for testing and treatment recommendations. On April 19, MTDPHHS requested assistance from CDC and the Food and Drug Administration (FDA).

## Investigation and Findings

### Identification of Index Patients and Implicated Foods

Investigation of the two index patients revealed that both persons had consumed a special sushi roll containing salmon and morel mushrooms. Morels were a new menu ingredient and were the only ingredient unique to the special sushi roll, making it an early suspected source of the outbreak. The morels were prepared in various ways during March 27–April 17. On April 8, morels were served partially cooked: a hot boiled sauce was poured over the raw morels, after which they were marinated for 75 minutes. On April 17, the morels were uncooked and cold-marinated before serving. During an inspection of restaurant A on April 18, food samples were collected, including salmon and morel pieces remaining from the original packaging. Multiple violations were identified at the time of inspection, including temperature control issues, improper time control and sanitization procedures, and improper storage of personal items. On the basis of the thorough environmental inspection and collection of food samples, clinical presentation of index patients, and early evidence implicating a specific food item, investigators determined that environmental sample collection was not necessary. On April 19, restaurant A informed GCCHD that it had received four additional telephone calls from patrons reporting illness after consumption of the special sushi roll. Other patrons reported illness directly to GCCHD throughout the investigation.

### Clinical Characteristics of Ill Persons

After a May 3 press release providing an update on the outbreak and soliciting additional cases, GCCHD continued to receive reports of gastrointestinal illness related to eating at restaurant A between March 28 and April 17. In total, the investigation identified 51 cases of illness* (including five cases among employees and 46 among restaurant patrons); of these cases, four ill persons visited a hospital emergency department, three were hospitalized, and the two index patients died (including one who sought emergency care but was not hospitalized). Among the 51 ill persons, 45 (88%) reported consumption of morels at restaurant A. Review of medical records for the three persons who were hospitalized and one person who sought emergency medical care without hospitalization revealed that the onset of gastrointestinal symptoms was rapid, with a median symptom onset of 1 hour after the restaurant meal. Vomiting and diarrhea were reportedly profuse, and hospitalized patients had clinical evidence of dehydration. The two patients who died had chronic underlying medical conditions that might have affected their ability to tolerate massive fluid loss.

### Testing of Clinical and Food Specimens

MTDPHHS and GCCHD coordinated with FDA for food specimen testing and with CDC’s toxicology and enteric diseases groups for clinical specimen testing.^†^ Food specimens were tested for multiple substances, including volatile and nonvolatile organic compounds and enteric pathogens.^§^ Neither clinical testing nor food testing identified a causative agent.

### Traceback of Implicated Food Items

Health department sanitarians traced the morels to a single importer, and a separate distributor that supplied mushrooms to multiple states. The morel mushrooms were cultivated and imported fresh from China. FDA and the California Department of Public Health contacted 12 California facilities that received morels from the same importer during January 1–May 17, 2023, six of which responded to the inquiries and reported receiving no illness complaints from patrons who ate morels prepared and served by those facilities. All six facilities reported cooking or otherwise thoroughly heating the morels before they were served. A CDC Epidemic Information Exchange (Epi-X) multistate call for cases did not result in additional case reports.

### Matched Case-Control Study

To identify foods associated with illness and assess a possible dose-response relationship, MTDPHHS and GCCHD, with support from CDC, conducted a matched case-control study. A questionnaire solicited demographic and symptom information and food items consumed.^¶^ A case was defined as the occurrence of diarrhea, nausea, vomiting, or abdominal pain in a person any time after eating at restaurant A during March 27–April 18, 2023. Cases were identified through reports received by restaurant A, public health departments, and case-patient interviews. Control participants were identified during case-patient interviews as dining partners who did not report illness after eating at restaurant A. Case-patients (or a proxy) and control participants were interviewed during May 4–12, 2023.

State and county health department staff members collected questionnaire data using Jotform software (version 4.0; Jotform Inc.). Deidentified data were sent to CDC for analysis. CDC personnel conducted matched and unmatched logistic regression analyses using SAS software (version 9.4; SAS Institute). This activity was reviewed by CDC, deemed not research, and was conducted consistent with applicable federal law and CDC policy.**

A total of 41 case-patients^††^ and 22 control participants responded to the questionnaire and were included in analyses, representing 29 unique dining parties. Overall, 27 (36.9%) case-patients were female and 31 (75.6%) were aged 20–49 years. A majority reported diarrhea (37; 90.2%), nausea (34; 82.9%), abdominal pain (28; 68.3%), loss of appetite (25; 61.0%), fatigue (24; 58.5%), vomiting (22; 53.7%), and abdominal distension (22; 53.7%) (Table 1).

**TABLE 1:** Characteristics of case-patients and control participants who dined at restaurant A — Montana, May 2023

Characteristic	No. (%)	
	Case-patients n = 41	Control participants n = 22
**Sex**		
Female	27 (65.9)	12 (54.5)
Male	14 (34.1)	10 (45.5)
**Age group, yrs**		
<20	0 (—)	4 (18.2)
20–49	31 (75.6)	12 (54.5)
≥50	10 (24.4)	6 (27.3)
Signs and symptoms*		
Diarrhea	37 (90.2)	NA
Nausea	34 (82.9)	NA
Abdominal pain	28 (68.3)	NA
Loss of appetite	25 (61.0)	NA
Fatigue	24 (58.5)	NA
Vomiting	22 (53.7)	NA
Abdominal distension or bloating	22 (53.7)	NA
Lightheadedness	18 (43.9)	NA
Dizziness	16 (39.0)	NA
Headaches^†^	15 (37.5)	NA
Chills	14 (34.1)	NA
**Other characteristics and outcomes**		
Hours from meal to symptom onset, median, range^†^	1 (0.25–90.00)	NA
Hours from meal to symptom resolution, median, range^†^	24 (1.00–672.00)	NA
Contacted a physician for symptoms	6 (14.60)	NA
Hospitalized for symptoms	3 (7.30)	NA
Deceased^§^	1 (2.40)	NA

**Abbreviation**: NA = not applicable.

* Reported by at least one third of case-patients. Case-patients could report multiple signs or symptoms.

^†^ Data missing for one case-patient.

^§^ One additional case-patient died but was not included in the case-control study because no proxy was available.

In logistic regression analyses matched on dining party, the odds of eating the special sushi roll with morels among case-patients were 15.78 times higher than the corresponding odds among control participants (Table 2). In addition, the odds of eating any morels among case-patients were 10.77 times higher than the corresponding odds among control participants. Other foods did not appear to be associated with case status.

**TABLE 2:** Exact logistic regression models for associations between consumption of menu items and case-patient status of restaurant A patrons — Montana, May 2023

Exposure	No. of case-patients n = 41	No. of control participants n = 22	OR (95% CI)		
			Matched analysis	Unmatched analysis	
			Model 1*	Model 2^†^	Model 3^§^
**Any morels**					
Yes	36	10	10.77 (1.37 to 492.84)	8.28 (2.12 to 37.80)	14.60 (2.75 to 151.00)
No	5	12	1.00 (Ref)	1.00 (Ref)	1.00 (Ref)
**Any special roll^¶^**					
Yes	33	8	15.78 (3.11 to ∞)**	6.95 (1.97 to 27.20)	34.30 (4.17 to >1,000)
No	8	14	1.00 (Ref)	1.00 (Ref)	1.00 (Ref)
**No. of pieces of special roll**					
None	8	14	1.00 (Ref)	1.00 (Ref)	1.00 (Ref)
1–3	9	7	0.50 (0.03 to ∞)**	2.20 (0.50 to 10.24)	18.06 (1.34 to >1,000)
≥4	24	1	22.47 (4.34 to ∞)**	38.15 (4.57 to >1,000)	248.46 (31.25 to ∞)**
**Odds per each additional piece of special roll consumed**	—	—	2.88 (1.34 to 16.90)	2.06 (1.44 to 3.23)	4.99 (2.06 to 24.95)

**Abbreviations**: OR = odds ratio; Ref = referent group.

* Conducted using conditional logistic regression models (matched on dining party). Only matched dining parties with at least one case-patient and one control participant contributed to the analysis; therefore, the numbers in the table do not match those in the matched analysis.

^†^ Unadjusted model.

^§^ Model adjusts for meal date (April 8–17, 2023 versus other) and age (continuous).

^¶^ Special roll is a sushi roll prepared with morel mushrooms.

** Indicates a median unbiased estimate.

When the amount of the special sushi roll consumed was modeled continuously, the association was positive between a one-piece increase in consumption of the special sushi roll and case status in all analyses (Table 2). When the special sushi roll was modeled as a three-level categorical variable (none, one–three pieces of a roll, and four pieces of a roll or more), associations in the unmatched models also suggested a dose-response relationship.^§§^

In models stratified by meal date, odds ratios comparing the odds of eating the special sushi roll (yes versus no) among 27 case-patients and 18 control participants were higher among those who ate at the restaurant on April 17, when uncooked morels were served, relative to April 8, when the morels reportedly underwent a partial cooking process (Table 3).

**TABLE 3:** Associations* between consumption of special sushi roll and restaurant A patron case-patient status, by meal date — Montana, May 2023

Meal date	Consumed special roll^†^	No. of case-patients	No. of control participants	Unmatched analysis, OR^§^ (95% CI)
**Apr 8**	Yes	7	4	11.67 (0.67 to ∞)
	No	0	3	1.00 (—)
**Apr 17**	Yes	20	3	99.57 (8.60 to ∞)
	No	0	8	1.00 (—)

**Abbreviation**: OR = odds ratio.

* Based on the common OR and exact 95% CIs.

**^†^** Special roll is a sushi roll prepared with morel mushrooms.

**^§^** The logit estimators use a correction of 0.5 in every cell.

## Public Health Response

In response to this outbreak, FDA published information on morels and other mushrooms that are traditionally wild and foraged but can also be cultivated (*1*). MTDPHHS and GCCHD published a press release summarizing the investigation's findings and provided recommendations on how to properly store and prepare morels to reduce the risk for illness (*2*). Restaurant A reopened to the public on May 25 once it was determined that public health risk had been mitigated by addressing health code violations. Restaurant A elected to stop serving morels.

## Discussion

The findings from this investigation suggest that uncooked or undercooked morel mushrooms were the likely source of the outbreak. The epidemiologic study demonstrated a clear association between consumption of the special sushi roll with morels and gastrointestinal illness, including a dose-response relationship, and an apparent stronger association among persons who ate the morels on a day when the morels served by restaurant A were reportedly uncooked. The California Department of Public Health and FDA investigation also supported the potential impact of preparation method on health outcomes, because no gastrointestinal illnesses were reported among patrons who ate morels at facilities where they were cooked before serving.

The signs and symptoms reported by ill persons and documented in medical records, including gastrointestinal illness and dizziness, are consistent with those reported in association with consumption of improperly handled, prepared, or cooked morels. Previous reports have described gastrointestinal illness after consumption of morels, which were consumed raw or cooked to varying degrees, as well as neurologic symptoms, including cerebellar effects, and, in some cases, death (*3*–*6*).

Morels should be refrigerated at a temperature of ≤40°F (≤4.4°C), in breathable type packaging, such as a paper bag. Morels should be cooked thoroughly before consumption because cooking is likely to reduce toxin levels present in the mushrooms (*1*).

### Limitations

The findings in this report are subject to at least five limitations. First, this investigation could not determine the specific characteristic of the morels that caused the outbreak. Morel storage and preparation methods, in addition to the differences in cooking methods described, that were not identified during the outbreak investigation might have differed between April 8 and 17, and might have played a role in causing illness. Second, morel mushroom toxins are not well characterized; therefore, the presence of a specific toxin could not be confirmed through laboratory testing (*1*). Third, limitations inherent to the epidemiologic studies included the small sample size and the possibility of unidentified confounding by something closely associated with the morels. Fourth, the study could have been affected by differential exposure misclassification (e.g., if case-patients had better recall of what they ate compared with control participants). Finally, responses could have been biased by public knowledge that morels were the suspected cause of illness.^¶¶^

### Implications for Public Health Practice

The investigation of this outbreak demonstrated how a coordinated collaborative public health response including local, state, and federal agencies can preserve and promote public safety. These findings also highlight gaps in knowledge regarding morels that need further research to better understand how they affect human health, and to identify effective treatment for morel toxicity beyond supportive care. Morel mushrooms should be cooked before human consumption to mitigate their potential toxicity.

## Footnotes

* Initially, a case was defined as the occurrence of gastrointestinal illness or neurologic symptoms in a person after eating at restaurant A during March 27–April 18, 2023, and the interval between these dates was considered the potential exposure period. However, follow-up indicated that all ill persons ate at the restaurant during March 28–April 17, 2023.

† Clinical testing for persons with specimens available included urine amatoxin testing, random total urine arsenic level, stool gastrointestinal panel and culture, and standard clinical laboratory tests used for medical management. Results of laboratory testing did not identify a causative agent.

§ Salmon and morel mushroom specimens tested negative for various known toxins, heavy metals, pesticides, volatile and nonvolatile organic substances, and pathogens, including *Clostridium perfringens, Bacillus cereus*, *Staphylococcal enterotoxin,* and other common enteric pathogens. DNA sequencing identified the mushroom specimen as *Morchella sextelata,* a species of true morel.

¶ The questionnaire included details related to dining (e.g., date and foods and beverages consumed), new or worsened symptoms after dining, receipt of medical care for those symptoms, underlying medical conditions, and dining partners. The survey accounted for the amount of each food eaten, menu modifications, unlisted food items (e.g., daily specials, chef’s choice, and “secret menu” items) and whether morel mushrooms were included in any food items. A proxy dining partner was interviewed to obtain information on one of the case-patients who was deceased.

** 45 C.F.R. part 46.102(l)(2), 21 C.F.R. part 56; 42 U.S.C. Sect. 241(d); 5 U.S.C. Sect. 552a; 44 U.S.C. Sect. 3501 et seq.

†† Among the 42 case-patients eligible for the case-control study, two were lost to follow-up, and one was deceased with no available proxy to interview. Two control participants were later found to have met the case definition and were reclassified as case-patients.

§§ An employee cohort study was also completed but had a small sample size. Eleven employees were interviewed, four of whom reported illness that met the case definition. Employees were not included in the case-control study. Employees who reported eating more pieces of the special sushi roll were more likely to report illness (Cochran-Armitage trend test two-sided exact p-value = 0.033). Using exact logistic regression, the odds ratio per one piece of special sushi roll increase was 1.92 (95% CI = 0.97–6.77; p-value = 0.067).

¶¶ Early in the investigation, morel mushrooms were publicly discussed as being a suspected cause of the outbreak, through media and social media channels. GCCHD and MTDPHHS mentioned morels as being a suspected cause of the outbreak in a press release on May 3, which was before most of the interviews were conducted.
